# NK Cell-Mediated Recall Responses: Memory-Like, Adaptive, or Antigen-Specific?

**DOI:** 10.3389/fcimb.2020.00208

**Published:** 2020-05-14

**Authors:** Victoria Stary, Georg Stary

**Affiliations:** ^1^Department of Surgery, Medical University of Vienna, Vienna, Austria; ^2^Department of Dermatology, Medical University of Vienna, Vienna, Austria; ^3^Ludwig Boltzmann Institute for Rare and Undiagnosed Diseases, Vienna, Austria; ^4^CeMM Research Center for Molecular Medicine of the Austrian Academy of Sciences, Vienna, Austria

**Keywords:** memory NK cells, adaptive immunity, vaccine strategy, recall response, trained immunity

## Abstract

Mounting experimental evidence hints to an import role for natural killer (NK) cells in adaptive immune responses to pathogens. NK cells with adaptive features are heterogeneous and belong to different subsets according to their phenotype as well as the nature of their adaptive recall reactions. Three types of adaptive NK cell responses have been described: (i) NK cells with long-lived memory of multiple different haptens and viral antigens were described in murine liver tissue with a possible human counterpart; (ii) infection of human and mouse cytomegalovirus is associated with an expansion of NKG2C^+^ and Ly49H^+^ NK cells, respectively, that selectively recognize CMV-encoded peptides thereby facilitating recall responses; (iii) cytokine-stimulated NK cells respond to different stimuli with enhanced production of IFN-γ after re-stimulation. These exciting findings not only support the idea of NK cells with adaptive features, but define a novel field of harnessing memory NK cell subsets for therapeutic strategies.

## Introduction

Immunological memory is a hallmark of adaptive immunity with leukocytes recognizing a previously encountered antigen and facilitating a specific and rapid immune response. Therefore, immunologic memory has the following basic characteristics: (i) enhanced response upon re-challenge to pathogens/antigens that have been encountered before and (ii) long-lived memory cells, that can persist even without the continuous presence of an antigen (Janeway et al., 2001). Immunological memory has been traditionally regarded as a unique feature of the two classical arms of the adaptive immune system, namely B cell-derived antibodies and T cells (Raff, 1973). T cells and B cells use recombinase–mediated recombination of variable gene segments (RAG) to generate a multitude of T and B cell antigen receptors (Bassing et al., 2002). Activation of the receptor by the appropriate antigen triggers clonal selection, differentiation and expansion of short-lived effector cells and long-lived memory cells. In contrast to B and T cells, all other leukocytes, including natural killer (NK) cells, do not express RAG proteins, have a limited repertoire of germline encoded receptors for the identification of target cells and are therefore considered as innate immune cells (Janeway and Medzhitov, 2002). The traditional view included that innate immune cells rapidly respond to pathogens, are critical in controlling viral infections and participate in tumor immunosurveillance in a non-specific fashion, but are unable to differentiate into memory cells (Lanier, 2005, 2007).

The paradigm of B and T cells as the sole carriers of immunological memory has been challenged by accumulating evidence indicating that NK cells respond to certain antigens with features of adaptive immunity. NK cell subsets mediating recall responses are heterogeneous with diverse functional properties. They differ in their phenotype and are currently described in the literature under various terms and concepts, such as adaptive, memory, memory-like and antigen-specific NK cells. In this review we summarize and categorize the current understanding of NK cells with adaptive features.

## Antigen-Specific NK Cell Responses

The first evidence for NK cell-associated recall responses came from mice deficient in T cells and B cells developing antigen-specific immunological memory to haptens, small molecules that form immunogenic adducts with proteins (O'Leary et al., 2006). When mice lacking B and T cells were sensitized by applying the hapten to the skin, they still developed a measurable recall response within a few days. Re-challenge with the hapten that had been applied before induced ear swelling, while application of a different hapten to sensitized mice did not induce a contact hypersensitivity reaction, indicating antigen specificity of this phenomenon (O'Leary et al., 2006). Using antibody depletion, genetic alterations and adoptive transfer of purified NK cell subsets into naïve RAG^−/−^γc^−/−^ recipients (which lack all endogenous lymphocytes, including NK cells), hapten-specific memory was identified in a subset of NK cells residing in the liver that was absent from the spleen (O'Leary et al., 2006).

**Figure 1:**
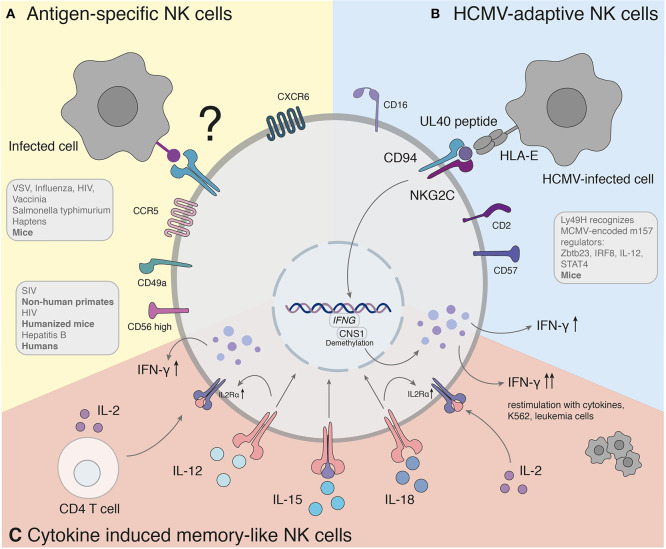
Antigen-specific recall responses of NK cell subsets. **(A)** Studies hint to the presence of a human counterpart of antigen-specific NK cells. The molecular mechanism of antigen-specific NK cells has yet to be found. **(B)** Adaptive NK cells in response to HCMV recognize variable UL40 peptides of different HCMV strains through NKG2C/CD94. **(C)** Cytokine induced memory-like NK cells activated with IL-12, IL-15, and IL-18 produce significantly more IFN-γ when re-stimulated with a non-specific stimulus. IFNG, Interferon-γ; CNS1, conserved non-coding sequence 1.

So far, different groups provided accumulating evidence that murine NK cells have adaptive immune features against various structurally different haptenized proteins [2,4-dinitro-1-fluorobenzene (DNFB), 4-ethoxy-methylene-2-phenyl-3-oxazalin-5-one (oxazolone) and picryl chloride] (Peng et al., 2013) as well as viral [VSV, influenza, HIV (Paust et al., 2010a), vaccinia virus (Gillard et al., 2011)] and bacterial [Salmonella typhimurium (Kupz et al., 2013)] antigens (Figure 1A).

Searching for antigen-specific memory NK cells in other species, Reeves et al. gave evidence for long-lived antigen-specific splenic and hepatic NK cells in SIV-infected rhesus macaques that lysed Gag- and Env-pulsed autologous dendritic cells compared to uninfected macaques. Blocking of the inhibitory NKG2A and activating NKG2C receptors significantly reduced antigen-specific killing, which suggested that these receptors play a role in antigen-specific target cell lysis. However, the contribution of NK cell receptors to antigen-specific responses needs to be clarified. NK cells from vaccinated animals demonstrated antigen-specific recall responses up to 5 years after vaccination (Reeves et al., 2015), indicating that antigen-specificity of NK cells might be relevant in primate species including humans.

Previous studies have long suggested that NK cell responses in humans might be antigen-specific. In HIV-1-exposed seronegative persons, increased NK cell anti-viral functions have been associated with protection (Scott-Algara et al., 2003; Ravet et al., 2007). In addition, potent NK cell responses in uninfected infants of HIV-1-positive mothers were linked with blockage of transmission *in utero* (Tiemessen et al., 2009), which hinted to pre-sensitization to the virus.

Subsequently, phenotypic analyses of human hepatic NK cells were suggestive for NK cell subsets similar to liver-derived NK cells in earlier animal studies (Marquardt et al., 2015; Stegmann et al., 2016). NK cells account for ~30–40% of all lymphocytes in human livers compared to relatively low NK cells frequencies in the peripheral blood (5–15%) which could be indicatory for a pool of a tissue-resident NK cell subset (Doherty et al., 1999). Human liver-resident NK cells are phenotypically different to blood-derived NK cells with increased expression of the subunit CD49a of the α1β1 integrin receptor. Hepatic CD49a^+^ NK cells resemble an immature phenotype with high expression of CD56, and low-to-absent expression of CD16 and CD57 (Marquardt et al., 2015; Stegmann et al., 2016). This is in contrast to the majority of blood NK cells that are characterized as CD56^dim^, CD57^+^, CD16^+^, Killer Ig-Like Receptor (KIR)^+^ cells and lack CD49a (Bjorkstrom et al., 2010). The heterogeneity of NK cell subsets is also reflected by the expression of T-box transcription factor (T-bet) and Eomesodermin (Eomes). Both transcription factors are crucial for specific developmental stages of NK cells (Gordon et al., 2012; Collins et al., 2017). NK cells isolated from human peripheral mononuclear cells are T-bet^hi^ and Eomes^low^ in spite of hepatic NK cells expressing low levels of T-bet (Knox et al., 2014; Stegmann et al., 2016). However, their function could not be directly linked to memory until a recent study demonstrated antigen-specific recall responses of NK cells in a humanized mouse model. These NK cells exhibit a phenotype similar to memory NK cells in blisters of individuals after re-exposure with peptides of varicella zoster virus (Nikzad et al., 2019). The observations of this study support mouse models of antigen re-challenge suggesting liver-resident NK cells to be capable to elicit antigen-specific recall responses in effector sites such as the skin. According to a recently published study human blood-derived NK cells exhibit antigen-specific cytotoxicity upon vaccination against or infection with hepatitis B (Wijaya et al., 2020). However, it is unclear whether (i) there is a well-defined subset of NK cells that is distinct in function and phenotype and (ii) this NK cell subset originates in the liver and appears in the blood stream *en route* to effector sites, as proposed previously (Paust et al., 2010b). Among NK cell lineages, liver-resident and skin-infiltrating NK cells appear to be highly related (Sojka et al., 2014). If liver NK cells differentiate from circulating precursor or have the ability to maintain and proliferate on site from progenitors that seeded in embryogenesis still needs to be proven (Peng et al., 2013; Cuff et al., 2016). Certain chemokine receptors have been shown to be important for homeostasis of hepatic NK cells. CXCR6 and CCR5 are mostly found on human liver NK cells and are largely absent from peripheral NK cells (Hudspeth et al., 2016; Stegmann et al., 2016). The corresponding ligands CXCL16, CCL3, and CCL5 are highly expressed by Kupffer cells, T cells, NK cells and endothelial cells on liver sinusoids (Heydtmann et al., 2005; Hudspeth et al., 2016). Additionally, hepatic CD56^bright^ NK cells can migrate in response to CCL3, CCL5, and CXCL16 (Hudspeth et al., 2016). However, direct proof of a liver-effector site axis and the molecular mechanism of recognition of various antigens remain to be identified.

## Adaptive NK Cells in CMV Infection

Recognition of target cells by NK cells is regulated through a variety of activating and inhibitory receptors. Ly49H is responsible for direct recall responses and subsequent resistance of mouse cytomegalovirus infection (MCMV) in certain strains, including C57BL/6 mice. As an activating receptor, Ly49H can engage the MCMV-encoded cell-surface glycoprotein m157 (Brown et al., 2001; Arase et al., 2002; Smith et al., 2002) (Figure 1B). Upon infection with MCMV, Ly49H^+^ NK cells undergo rapid clonal proliferation followed by a contraction phase, which was not observed in other viral infections (Daniels et al., 2001; Dokun et al., 2001). Adoptive transfer of Ly49H^+^ NK cells 50 days after infection was capable to induce a robust secondary expansion und enhanced effector function upon re-challenge in naïve mice (Sun et al., 2009). The transcription factor Zbtb23 is upregulated in NK cells upon MCMV infection and crucial as regulator for the proliferation machinery of MCMV-adaptive NK cells by antagonizing the anti-proliferative factor Blimp-1 (Beaulieu et al., 2014). IL-12 receptor-deficient NK cells fail to expand after MCMV infection and do not mediate protection after re-challenge (Sun et al., 2012), indicating that proinflammatory cytokine signaling is required for the generation of adaptive NK cells toward MCMV. Subsequently, IL-12 and STAT4 promote epigenetic remodeling of the IRF8 locus and therefore upregulation of IRF8. Furthermore, IRF8 is associated with the proliferative burst of adaptive NK cells (Adams et al., 2018).

NKG2C is the human counterpart of the murine Ly49H (Sun et al., 2011) (Figure 1B). The infection of human cytomegalovirus is associated with an increased expression of HLA-E binding and activating the NK cell receptor NKG2C (Guma et al., 2004). An expansion of NKG2C^+^ NK cells was observed during CMV infection (Guma et al., 2006a), similar to the response of Ly49H^+^ NK cells in MCMV. NKG2C^+^ NK cells of CMV-seropositive donors persist at higher frequencies and exhibit enhanced activity in response to CMV reactivation compared with NKG2C^+^ NK cells from seronegative donors (Lopez-Verges et al., 2011; Foley et al., 2012). In the case report of a 3 year-old patient with T^−^B^+^NK^+^ severe combined immunodeficiency (SCID), NKG2C^+^ NK cells were the sole provider of protective immunity against CMV after significant expansion of IFN-γ-producing CD16^+^CD94^−^NKG2C^+^ NK cells (Kuijpers et al., 2008). NK cells with adaptive features to HCMV have been furthermore identified in peripheral blood, liver and lung of seropositive individuals (Guma et al., 2004; Beziat et al., 2012; Marquardt et al., 2015, 2019). Notably, a similar expansion of NKG2C^+^ NK cells in HCMV-seropositive individuals has also been observed during infections with other pathogens, such as the Hantavirus (Bjorkstrom et al., 2011), HIV (Guma et al., 2006b), EBV (Saghafian-Hedengren et al., 2013) and malaria (Hart et al., 2019).

Besides NKG2C expression, HCMV-adaptive NK cells display a distinct receptor profile with a more mature and differentiated phenotype, including upregulation of CD57 and decreased expression of the inhibitory receptor NKG2A. This subset also expresses killer cell immunoglobin-like receptor (KIR) specific for self-MHC (Guma et al., 2004; Beziat et al., 2012; Schlums et al., 2015). Recent studies reveal stable alterations of transcriptional programs of HMCV-adaptive NK cells. This includes epigenetic imprint of the INFG locus, which drives IFN-γ expression by HCMV-adaptive NK cells (Luetke-Eversloh et al., 2014). In a cohort of 196 healthy adults, HCMV seropositivity was correlated with a lack of FcεRγ, SYK, and EAT-2 expression in CD56^dim^ NK cells of the peripheral blood which correlated with a downregulation of the transcription factor ZBTB16 (Zinc Finger and BTB Domain Containing 16). ZBTB16 was shown to bind the promotors of these genes (Schlums et al., 2015). Preferential expansion upon HCMV was largely confined to FcRγ-deficient NK cells in an antibody-dependent fashion (Lee et al., 2015). The mechanism of HCMV-adaptive NKG2C^+^ NK cells in the blood of patients with a history of HCMV infection was ultimately proven to depend on the recognition of UL40 encoded HCMV peptides, which stabilize and load on HLA-E by NKG2C (Hammer et al., 2018; Rolle et al., 2018). Interestingly, NKG2C^+^ NK cells were able to differentially discriminate HCMV strains encoding for variable UL40 peptides.

There is emerging interest in the metabolic regulation of NK cell cytotoxicity in viral infections including adaptive immune functions. In general, naïve NK cells rely on glucose fueled by oxidative phosphorylation (Gardiner, 2019). Inhibition of glycolysis, however, decreases the clearance of MCMV-infected murine target cells by Ly49H^+^ NK cells indicating that NK cells require glycolysis for cytotoxicity during viral infection (Mah et al., 2017). Studies with KIR^+^ educated blood-derived NK cells showed increased rates of glycolysis compared to uneducated NK cells even in a resting state (Pfeifer et al., 2018; Schafer et al., 2019). It was furthermore reported that CD56^bright^ NK cells express high levels of the glucose uptake receptor Glut1 and have therefore increased rates of glucose intake compared with CD56^dim^ NK cells (Keating et al., 2016). The elevated glycolytic metabolism of CD56^bright^ NK cells would consequently support the energy demands of increased interferon-gamma (IFN-γ) production in the event of an immune response. In a cohort of HCMV seropositive individuals, NKG2C^+^CD57^+^ NK cells exhibited increased oxidative and glycolytic metabolic profiles compared to seronegative donors. Moreover, this NK cell subset of HCMV-experienced individuals also demonstrated enhanced mitochondrial membrane potential, higher expression of genes associated with the mitochondrial ATP synthase production and electron transport chain (Cichocki et al., 2018).

However, studies give hints to the existence of NKG2C-independent pathways of HCMV-adaptive NK cell differentiation (Muntasell et al., 2016) or emphasize a contribution of certain activating KIRs in addition to NKG2C (Beziat et al., 2012). Della Chiesa et al. studied patients with hematological malignancies after umbilical cord blood transplantation of donors carrying a homozygous deletion of the NKG2C gene. Although NKG2C was missing, they described an expansion of CD56^dim^NKG2A^−^KIR^+^ NK cells in response to CMV infection (Della Chiesa et al., 2014). These reports may be an indicator of possibly different mechanism other than NKG2C being able to facilitate recall responses against CMV.

## Cytokine-Induced Memory-Like NK Cells

In 2009, Cooper et al. demonstrated that murine NK cells activated with cytokines produce significantly more IFN-γ when re-stimulated after up to 22 days of prior activation with IL-12, IL-15, and IL-18. Pre-activated NK cells elicit an enhanced effector function against tumor cell lines. It was further shown that memory-like “trained” features are passed on to the next non-activated generation of NK cells (Cooper et al., 2009). Since the observed adaptive responses arise as a result of non-specific activation, the term cytokine-induced memory-like (CIML) NK cell was formed. CIML NK cells display some hallmarks of adaptive immunity, as they are long-lived, exhibit enhanced anti-tumor as well as anti-viral immune responses, and have certain epigenetic modifications.

Several features of CIML NK cells hold true for humans (summarized in Figure 1C). Analogous to NK cells of mice, human NK cells activated with cytokines were described to increase IFN-γ production after re-stimulation with cytokines, K562, or leukemia cells (Romee et al., 2012, 2016). The mechanisms underlying the effect of these cytokines are incompletely understood. Studies of the contributions of each cytokine (IL-12, IL-15, and IL-18) demonstrated a synergistic effect in degranulation, IFN-γ, TNF-α, and CCL3 production. At the same time IL-15 alone or in combination exhibited the highest cytotoxicity against K562 (Terren et al., 2018). The NK cell intrinsic ability for IFN-γ production coincided with demethylation of the conserved non-coding sequence (CNS) 1 in the *Ifng* locus, enhancing transcription of *Ifng* and was additionally maintained through IL-2 production of CD4^+^ T cells (Ni et al., 2016). The enhanced NK cell proliferation and cytotoxicity of CIML NK cells arose due to a prolonged expression of IL2Rα (CD25) resulting in a high responsiveness of IL-2 receptor stimulation (Leong et al., 2014). In addition, IL-12 and IL-18 were shown to upregulate the IL2Rα chain sensitizing NK cells to IL-2 stimulation (Lee et al., 2012). In NK cells the upregulation of the mammalian target rapamycin complex 1 (mTORC1) pathway was associated with the acquisition of NK cell effector functions (Donnelly et al., 2014). IL-15 and IL-18 can activate mTORC1, which plays a key role for glycolytic reprogramming and facilitated upregulation of glycolytic enzymes in NK cells (Marcais et al., 2014; Almutairi et al., 2019).

These features of CIML NK cells represent a potential approach for translational immunotherapy. CIML NK cells promoted enhanced anti-tumor activity after adoptive transfer in a melanoma xenograft model (Ni et al., 2016). Adoptive transfer of donor NK cells demonstrated to mediate graft-vs.-leukemia effects while suppressing acute graft-vs.- host disease in a murine model of allogeneic hemopoietic stem cell transplantation (Song et al., 2018). Results from a first-in-human phase 1 clinical trial with adoptively transferred CIML NK cells exhibited increased effector functions against leukemia targets leading to favorable clinical responses and remissions in a subset of AML patients (Romee et al., 2016).

## Conclusion

The findings summarized in this review suggest that NK cells are able to fundamentally change the way they respond to later activation, resulting in features which, in certain subsets, fulfill the requirements of immunologic memory or, at least, a trained immune response. The discovery of adaptive NK cells gives rise to many questions. For instance, what are the precise mechanism of development of different subsets of NK cells? What molecular pathways underlie the specificity to a variety of antigens? Which factors drive NK cell activation, proliferation and generation of immunological memory? How are long-lived memory NK cells maintained? The paradigm shift of NK cells from classical innate killers to sophisticated immune cells possessing attributes of both entities has just begun. Further investigations into NK cell memory will have major implications in vaccination or immunotherapeutic approaches.

## Author Contributions

VS and GS wrote the manuscript. VS created the figure with BioRender.

## Conflict of Interest

The authors declare that the research was conducted in the absence of any commercial or financial relationships that could be construed as a potential conflict of interest.
